# A Deep Learning Approach to Vascular Structure Segmentation in Dermoscopy Colour Images

**DOI:** 10.1155/2018/5049390

**Published:** 2018-11-01

**Authors:** Joanna Jaworek-Korjakowska

**Affiliations:** Department of Automatic Control and Robotics, AGH University of Science and Technology, Cracow, Poland

## Abstract

**Background:**

Atypical vascular pattern is one of the most important features by differentiating between benign and malignant pigmented skin lesions. Detection and analysis of vascular structures is a necessary initial step for skin mole assessment; it is a prerequisite step to provide an accurate outcome for the widely used 7-point checklist diagnostic algorithm.

**Methods:**

In this research we present a fully automated machine learning approach for segmenting vascular structures in dermoscopy colour images. The U-Net architecture is based on convolutional networks and designed for fast and precise segmentation of images. After preprocessing the images are randomly divided into 146516 patches of 64 × 64 pixels each.

**Results:**

On the independent validation dataset including 74 images our implemented method showed high segmentation accuracy. For the U-Net convolutional neural network, an average DSC of 0.84, sensitivity 0.85, and specificity 0.81 has been achieved.

**Conclusion:**

Vascular structures due to small size and similarity to other local structures create enormous difficulties during the segmentation and assessment process. The use of advanced segmentation methods like deep learning, especially convolutional neural networks, has the potential to improve the accuracy of advanced local structure detection.

## 1. Introduction

**Figure 1:**
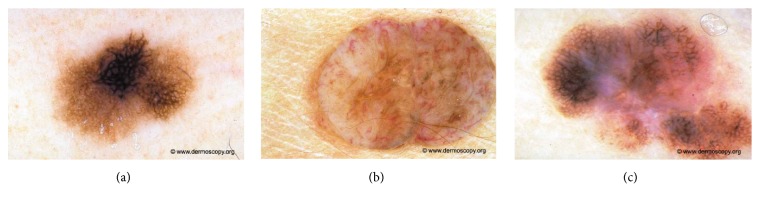
Melanocytic lesion analysis is one of the most difficult areas in modern dermatology. The challenges fall into two broad categories, namely, the recognition of rare but characteristic entities and the much more common problem of where to place an unusual lesion on the spectrum of melanocytic lesions. Examples of melanocytic lesions: (a) melanoma in situ, (b) dermal nevus, Unna type, and (c) early invasive melanoma.

Melanoma is the deadliest form of skin cancer which develops when skin cells multiply rapidly as a consequence of mutations in their DNA caused by the sun's ultraviolet (UV) radiation (Figure 1). Melanoma is a cancer that starts in the melanocytes which are cells that make a brown pigment called melanin, which gives the skin its tan or brown colour [1]. Other names for this cancer include malignant melanoma and cutaneous melanoma. Most melanoma cells still make melanin, so melanoma tumours are usually brown or black but can appear pink, tan, or even white [1]. The introduction of dermoscopy has improved the accuracy of diagnosis of melanoma.

According to the World Health Organization about 132,000 new cases of malignant melanoma are diagnosed worldwide each year. In some parts of the world, especially in New Zealand and Australia, melanoma is becoming more common every year and has more than doubled in the past 30 years [2]. For 2018 it is predicted that 14,320 new cases of melanoma skin cancer will be diagnosed in Australia which is estimated to be 10,4% of all new diagnosed cancer cases [3].

**Figure 2:**
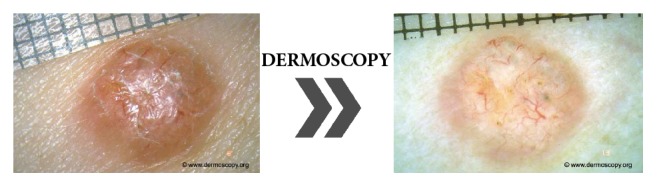
Examination of pigment mole with a dermatoscope. Diagnostic instrument commonly used for dermoscopic examination is a hand-held dermatoscope. Dermoscopy is a noninvasive method that allows evaluation of colours and microstructures of the epidermis, the dermoepidermal junction, and the papillary dermis not visible to the naked eye.

One of the main goals in prevention of malignant melanoma is awareness, early diagnosis and surgical excision. The introduction of dermoscopy has improved the accuracy of diagnosis of melanoma. Digital dermoscopy is currently the most used technology, although novel methods, such as confocal microscopy, show promising result. Nowadays, dermatologists work in order to define the different skin lesion types based on dermatoscopic features to improve early detection (Figure 2) [4].

### 1.1. Motivation

**Figure 3:**
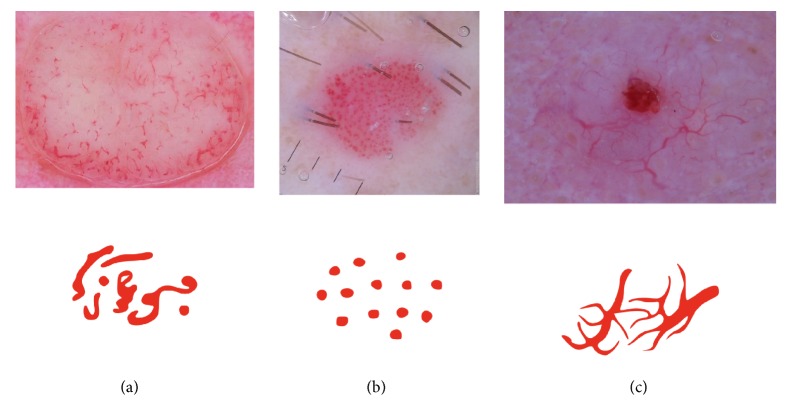
Examples of vascular patterns that can be identified in melanocytic lesions: (a) polymorphous vessels: different vascular morphologies in the same mole, (b) dotted vessels: small, reddish vessels that resemble a pinhead, and (c) arborizing vessels: large vessels that branch into finer, small vessels [5].

Recently, the most commonly used diagnostic method is the 7-point checklist algorithm. The 7-point checklist is a diagnostic method that requires the identification of only 7 dermoscopic criteria, thus enabling even less experienced clinicians to perform the skin mole examination. One of the most important dermoscopic criteria is the atypical vascular pattern which has the second highest odds ratio of 7.42 and is among the 3 most important features that indicate the malignancy of the skin mole. Atypical vascular pattern appears as linear-irregular or dotted vessels not clearly combined with regression structures and associated with other local structures [6, 7]. Dermoscopy images show only the horizontal inspection of the skin lesion. Vascular structures that are located in parallel to the skin's surface will appear as a line, while those located vertically to the skin's surface will become visible as dots and nodes. In this respect, we observe a strong connection between the vascular structure and tumour progression and volume. Flat and superficial amelanotic/hypomelanotic melanoma and basal cell carcinoma will display different vascular structures than those of their thick or nodular counterparts (Figure 3) [8].

Based on the information above exact detection and classification of vascular structures is a crucial step in early and accurate diagnosis of malignant melanoma. In this paper we present a new and of the first approaches to the detection of vessels in dermoscopic colour images. In this study, deep learning methods (CNNs) have been used to fully automatically localize and segment vascular structure. To evaluate the performance of deep learning segmentation, we compared the outcome with the manual segmentation. Deep learning technologies have the potential to improve the accuracy and speed up the diagnosis of skin structure in clinical settings.

### 1.2. Related Works

To the best of our knowledge only few attempts have been made to detect vascular structures in dermoscopic colour images. Recently, Kharazmi et al. in work [9] proposed a data-driven feature learning framework based on stacked sparse autoencoders (SSAE) for comprehensive detection of cutaneous vessels. The proposed framework demonstrated performance of 95.4% detection accuracy over a variety of vessel patterns. Betta et al. in work [10] presented a method for the identification of atypical vessels. Due to the difficulty to obtain a relevant number of epiluminescence microscopy (ELM) images with the occurrence of this local structure, the training set was constituted by pixels selected as vascular pattern in a set of images containing occurrences of this criterion. The Hue, Saturation, and Luminance components were evaluated and the frequency histograms corresponding to the three colour planes were determined. The pixel classification depended on the value of the particular HSL component. However, the authors warned that in some cases the algorithm misclassified the area, evidencing a low specificity [4, 11]. In 2014, Fabbrocini et al. proposed an automatic detection algorithm combining colour segmentation and structural analysis [12]. The skin lesion area was matched with the texture descriptors (entropy, inverse difference moment, and correlation) based on the gray level cooccurrence matrix in order to exclude texture areas. Then, a statistical analysis of the segments was performed. The system has been tested on 200 medical images and achieved 80% sensitivity, and 78% specificity. More recently Kharazmi et al. [13] proposed a new approach to vessel segmentation. Authors firstly decompose the image using independent component analysis into melanin and hemoglobin. Using k-means clustering, the hemoglobin component is clustered and a vessel mask is generated as a result of global thresholding. The segmentation sensitivity and specificity of 90% and 86% were achieved on a set of 500 000 manually segmented pixels provided by an expert. Recently, advances have been observed in retinal vessel segmentation, which is another medical area, where vessel segmentation is crucial for accurate diagnosis and early treatment. In [14] authors present the implementation of the neural network structure derived from the U-Net architecture. The algorithm obtained an AUC-ROC accuracy of 0.98.

## 2. Material and Methods

Artificial intelligence research has been around for more than half a century but in recent years a huge progress is observed in widely understood machine learning [15]. Advanced statistical techniques, known as deep learning models, have been exploited with impressive results. Convolutional neural networks applied in this research learn feature representation automatically from the training data [16]. Deep learning in general and convolutional neural networks in particular have been used in variety of pattern recognition problems like retinal vessel segmentation, lung area detection, or breast cancer classification [17].

### 2.1. Overview

**Figure 4:**
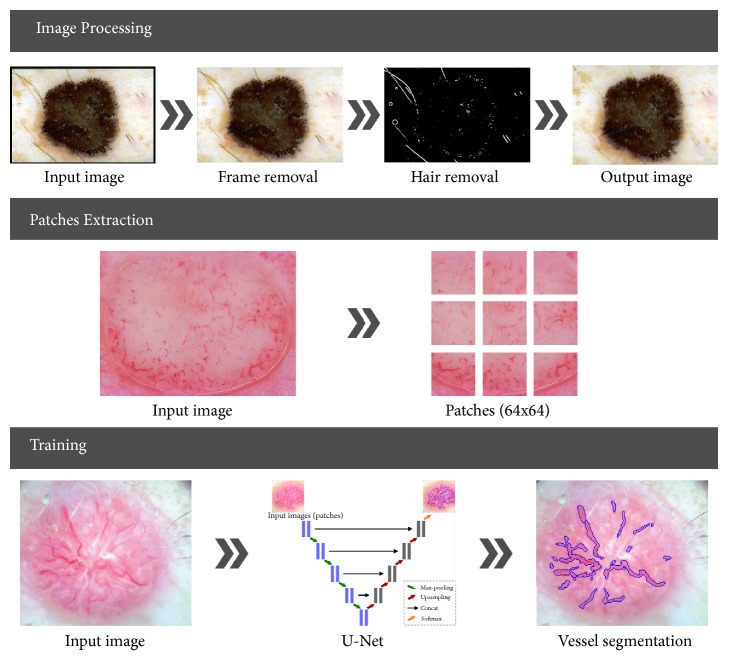
Illustration of the proposed system. (a) Image preprocessing involves black frame, hair and air bubble detection, removal or inpainting, and furthermore image normalization. (b) Patch extraction is carried out in a randomly sliding window with some overlap. (c) U-Net architecture construction and neural network training.

As illustrated in Figure 4 the implemented application is divided into four stages: preprocessing (image enhancement), patch extraction, training, and validation.

In this research we use the U-Net convolutional network architecture introduced by Ronneberger et al. in 2015 [18]. This method was chosen because it is one of the most promising for the addressed problem, especially, advanced image segmentation. In this section, the preprocessing step is described shortly, based on previous works, while the network architecture and training phase are presented in detail. The preprocessing and patch extraction stages have been implemented in Matlab 2017a while the neural architecture and classification process has been performed in Python using U-Net implementation as proposed by Ronneberger* et al.* and developed with TensorFlow [18, 19].

### 2.2. Image Preprocessing

The first step in every medical image processing system is the image acquisition, which aims at obtaining an image of the highest quality. After dermoscopic image is acquired, it may not have the expected quality to perform the diagnostic analysis. Dermoscopic images are inhomogeneous and complex and furthermore include extraneous artifacts, such as skin lines, air bubbles, and hairs, which appear in virtually every image. The preprocessing stage consists of two parts. The first step is the removal of black frame that is introduced during the digitization process. The second step is a hair-removal algorithm which comprises two parts: hair detection and inpainting. These steps have been precisely described in our previous works [20, 21].

### 2.3. Patches Extraction

After preprocessing we extract *N* small patches *x*_*m*,*n*_ of size 64 × 64 from the dermoscopy image *I* and the corresponding annotation *G* at the same position. The so-called ground-truth mask contains zeros and ones, where (*x*_*i*_, *y*_*j*_) = 1 informs that there is a vessel area at this location. Patch extraction can be performed in few different ways: nonoverlapping, overlapping, and randomly extracted. Each of these solutions has its advantages and disadvantages. To avoid the problem of class imbalance, patches have been extracted randomly around pixels pointing to vessel area both from dermoscopy image as well as from the accompanying masks. For 74 dermoscopy images with different resolution we have obtained 146516 patches. The size of the patches has been chosen experimentally and concatenated with the U-Net architecture. Larger patches require more max-pooling layers that reduce the localization accuracy, while small patches allow the network to see only little context [18].

### 2.4. Network Architecture

**Figure 5:**
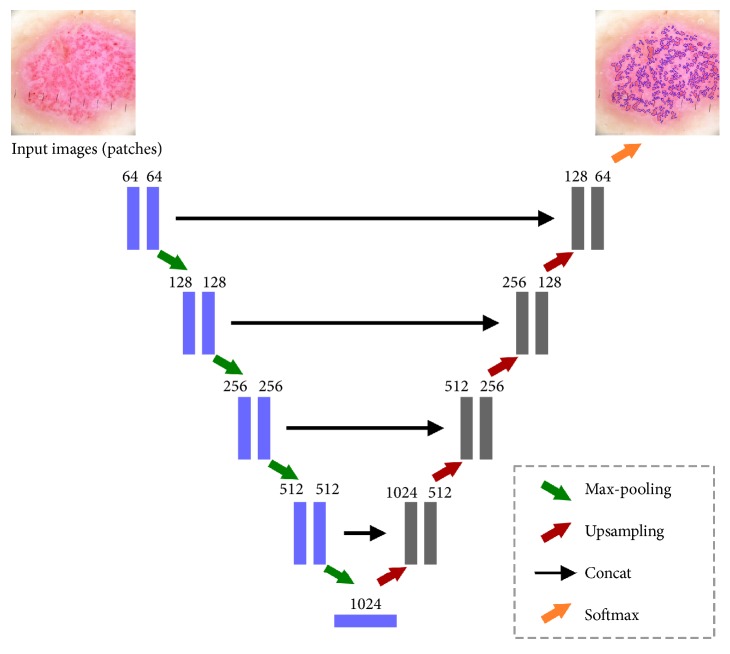
Original layers of U-Net: an encoder-decoder architecture. Each box corresponds to a multichannel feature map. The horizontal arrow denotes transfer residual information form early stage to later stage.

U-Net convolutional network is a popular architecture in the class of encoder-decoders, where the encoder reduces the spatial dimension of objects with pooling layers while decoder recovers the object details with upsampling layers. U-Net is a modified and extended version of fully convolutional network.  Figure 5 presents the overview of a U-Net architecture.

U-Net consists of contracting path (left side) and an expansive path (right side). U-Net is like a combination of convolutional and deconvolution layers. Contracting path structurally repeats a typical 3 × 3 convolutional layer (unpadded convolutions) followed by a Rectified Linear Unit and a 2 × 2 max-pooling operation with stride 2 for downsampling. On the expansive path, information is merged from layers of contracting path of appropriate resolution and layers of expansive path of lower resolution, so that a whole network recognizes patterns at several scales. The final layer of a 1 × 1 convolution is used to map each 64-component feature vectors to the desired number of classes [18]. Input image is firstly passed through a convolutional layer with Rectified Liner Unit (ReLu) activation function:

(1) $$\begin{array}{c} f\left(\;x\;\right)=\max\left(\;0,x\;\right) \end{array}$$

The rectifier is an activation function defined as the positive part of its argument where *x* is the input to a neuron. Our first layer has 64-feature map. Afterwards the max-pooling operation is applied. The convolutional layers downsample the spatial dimension from 64 × 64 to 8 × 8. The expansive path consists of an upsampling of the feature map followed by upconvolutional and convolution layers with ReLU [17]. As we have only 2 classes (present or absent) we use the softmax classifier that calculates the cross-entropy loss for every single example:

(2) $$\begin{array}{c} L_{i}=-\log\left(\;\frac{e^{f_{y_{i}}}}{\sum_{j}e^{f_{j}}}\;\right) \end{array}$$

The encoder and decoder comprise five layers with (64 - 128 - 256 - 512 - 1024) and (1024 - 512 - 256 - 128 - 64) filters of size (3 *x* 3) pixels, respectively. We have chosen the Adam optimization algorithm that is an extension to stochastic gradient descent that has recently seen broader adoption for deep learning applications in computer vision and natural language processing.

## 3. Results

### 3.1. Image Database

**Figure 6:**
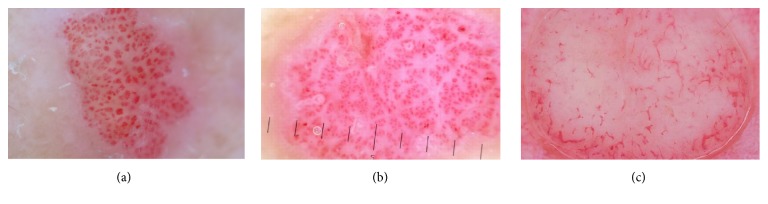
Database examples.

The proposed segmentation method based on the U-Net neural network architecture has been tested on colour dermoscopic images from a widely used Interactive Atlas of Dermoscopy [6]. Images for this repository have been provided by two university hospitals (University of Naples, Italy, and University of Graz, Austria) and stored on a CD-ROM in the JPEG format. The documentation of each dermoscopic image was performed using a Dermaphot apparatus (Heine, Optotechnik, Herrsching, Germany) and a photo camera (Nikon F3) mounted on a stereomicroscope (Wild M650, Heerbrugg AG, Switzerland) in order to produce digitized ELM images of skin lesions. All the images have been assessed manually by a dermoscopic expert with an extensive clinical experience. Additionally, colour images have been used from the *PH*^2^ database [22], and images available in online libraries [23, 24]. The database included 74 cases with different types of vascular pattern including linear, dotted, comma, hairpin, and polymorphous. Dermoscopy colour images have different resolutions, ranging from 0.033 to 0.5mm/pixels.  Figure 6 presents samples from our database.

### 3.2. Deep Network (CNN) Training

**Figure 7:**
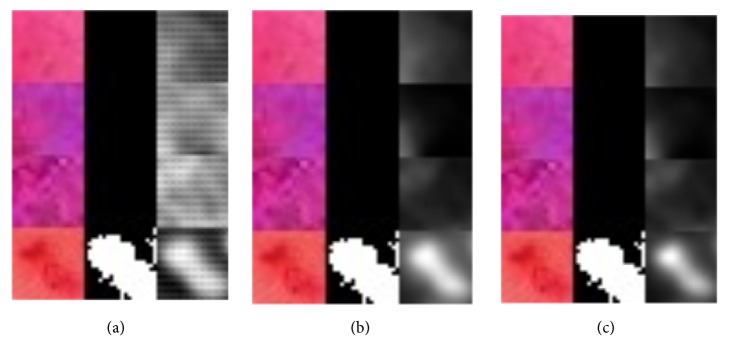
Visualization of segmentation precision over 100 epochs. Colour vascular structures in training patch slices (left), accompanying ground-truth masks (middle), and the predictions (right): (a) 10 epochs, (b) 76 epochs, and (c) 95 epochs.

The database set was divided into training set (80%) and test set (20%). The training set was used for train the U-Net, while the test set was used to analyse the training versus test error in case of overfitting. In the training stage the input image (patch) and the corresponding ground-truth mask are used to train the implemented U-Net network. The softmax layer at the end of the network creates a probabilistic two-channel output, just like a binary segmentation problem. However, the ground truth here is a probabilistic map, not a binary segmentation map. Training is performed for 100 epochs. As training continuous (seen by epoch) we can see that the generated mask becomes more precise (Figure 7). Grayscale images in Figure 7 present predictions as a grayscale map, where light colours display values near 1, while dark colours display values near 0.

**Figure 8:**
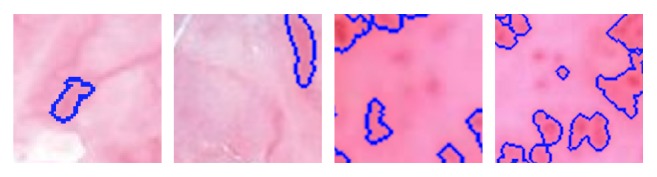
Examples of the incorrect segmentation of vascular structures. Different unmarked vascular patterns are still visible.

For the U-Net architecture the patch-size, the batch-size, and the weighted pixel-wise cross entropy were proved to be of high importance. The patch-size proved to be best at 64 × 64 pixels with a large batch-size. When the batch-size is too low, it might be unable to learn, thus negatively impacting total computation time. The predictions were thresholded at 0.5 and are displayed as a binary masks (Figure 8).

### 3.3. Analysis of CNN Segmentation Method

The performance of the U-Net neural network vascular structure segmentation approach can be assessed based on the analysis of* Sørensen index* also known as* dice similarity coefficient* which is a statistic used for comparing the similarity of two samples [25].

Given two binary sets, *X* and *Y*, the Sørensen's formula is defined as

(3) $$\begin{array}{c} DSC=\frac{2\left|X\cap Y\right|}{\left|X\right|+\left|Y\right|} \end{array}$$

Using the definition of true positive (TP), false positive (FP), and false negative (FN), it can be written as

(4) $$\begin{array}{c} DSC=\frac{2TP}{2TP+FP+FN} \end{array}$$

where TP denotes vascular structure pixels, FP denotes vascular structure pixels not detected, FP denotes background pixels classified as vascular structures.

The DSC is a statistical measure that calculates the degree of overlapping between the experimental segmentation and the manual segmentation and is frequently used to compare segmentations.

Furthermore, sensitivity and specificity are calculated using following equation:

(5) SE=TPTP+FN

(6) SP=TNTN+FP

The proposed algorithm achieved an average DSC of 0.84, sensitivity 0.85, and specificity 0.81. Possible values of DSC range from 0.0 to 1.0. A perfect classifier or segmentation model achieves a DSC of 1.0. The mean DSC scored 0.84 in range of 0.54-0.92. Taking into account the fact that the vascular structures have been segmented manually for the ground-truth mask the achieved DSC score is very promising. We observed that the algorithm misclassified areas which were on the boarder between skin lesion and vascular structures as well as the red surrounding between skin mole and healthy skin (Figure 8).

**Figure 9:**
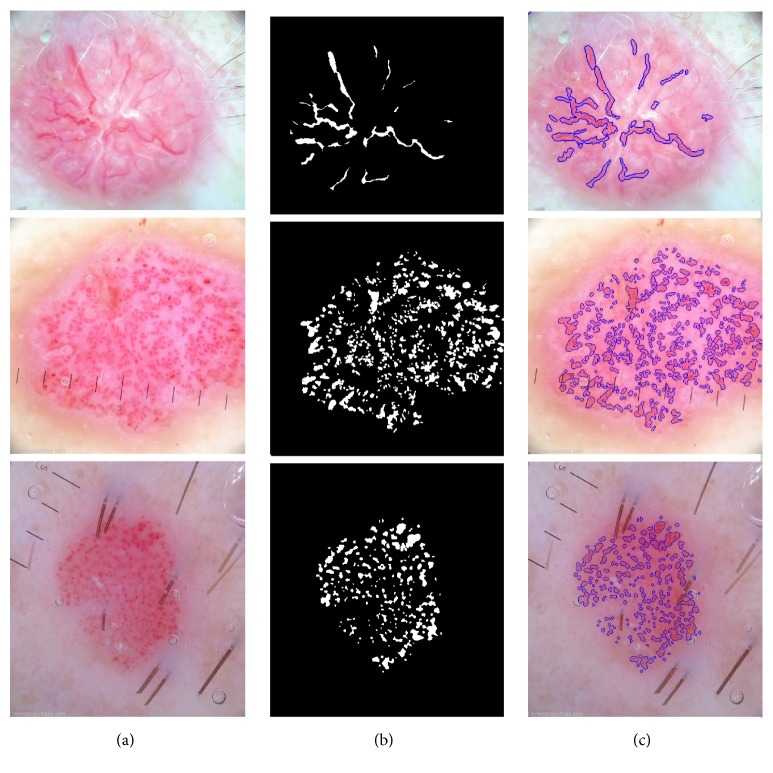
Example of dermoscopy images, accompanying segmentation masks and the predictions.

Figure 9 presents few segmentation results with corresponding ground-truth masks and the predictions of the previously unseen test data. The figure contains three columns. From the left to the right, each one represents the original image, the ground-truth, and the segmentation result using the generated map, respectively. Ground-truth masks and predictions have been inverted, where white represents vessel tissue and black nonvessel tissue.

Section* Related Works* presented the state of the art of previous studies concerning the segmentation of vascular patterns in dermoscopy images. In paper [13] authors presented a method that achieved sensitivity and specificity of 90% and 86%. However, it is difficult to compare these results because our method has been tested on a much broader database with more complex images. For the evaluation of the advantages and correctness of the implemented algorithm, we present several outcomes with segmented structures (Figure 9).

## 4. Conclusions

In this research we have obtained accurate and comprehensive results showing that the applying of U-Net neural networks for local structure detection in dermoscopy colour images brings a valuable alternative to vascular structure detection. We believe that this solution can be implemented as part of a vascular pattern classification algorithm or furthermore a computer-aided diagnostic system for early detection of melanoma. Our technique shows a clear advantage over other implemented and stated in the related works section algorithms including detection accuracy, insensitive to different dermoscopy image acquisition methods.

### 4.1. Discussion

Starting from the described framework, further research efforts will be firstly addressed to compare and integrate the very promising approaches reported in the most recent literature, in order to improve the neural network and optimize layers and parameters. We will also conduct a follow-up study after collecting more data with different vascular patterns. Future research will concentrate on the possibility of vascular structure classification.

## Data Availability

Previously reported dermoscopy images were used to support this study and are available at 10.1016/j.jaad.2003.07.029. These prior studies (and datasets) are cited at relevant places within the text as [6].
